# *Shigella flexneri* serotype 1c derived from serotype 1a by acquisition of *gtrIC* gene cluster via a bacteriophage

**DOI:** 10.1186/s12866-016-0746-z

**Published:** 2016-06-27

**Authors:** Swee-Seong Tang, Nils I. A. Carlin, Kaisar A. Talukder, Phung D. Cam, Naresh K. Verma

**Affiliations:** Division of Biomedical Science and Biochemistry, Research School of Biology, The Australian National University, Bldg #134 Linnaeus Way, Canberra, ACT 0200 Australia; Etvax AB, Gunnar Asplunds Allé 16, SE-171 63 Solna, Stockholm Sweden; International Centre for Diarrhoeal Diseases Research, Dhaka, Bangladesh; Department of Microbiology, National Institute of Hygiene and Epidemiology, Hanoi, Vietnam; Division of Microbiology, Institute of Biological Sciences, Faculty of Science, University of Malaya, 50603 Kuala Lumpur, Malaysia

**Keywords:** *Shigella flexneri*, Bacillary dysentery, Serotype-conversion, Evolutionary origin, Glucosyltransferase, Serotype 1c

## Abstract

**Background:**

*Shigella* spp. are the primary causative agents of bacillary dysentery. Since its emergence in the late 1980s, the *S. flexneri* serotype 1c remains poorly understood, particularly with regard to its origin and genetic evolution. This article provides a molecular insight into this novel serotype and the *gtrIC* gene cluster that determines its unique immune recognition.

**Results:**

A PCR of the *gtrIC* cluster showed that serotype 1c isolates from different geographical origins were genetically conserved. An analysis of sequences flanking the *gtrIC* cluster revealed remnants of a prophage genome, in particular integrase and tRNA^Pro^ genes. Meanwhile, Southern blot analyses on serotype 1c, 1a and 1b strains indicated that all the tested serotype 1c strains may have had a common origin that has since remained distinct from the closely related 1a and 1b serotypes. The identification of prophage genes upstream of the *gtrIC* cluster is consistent with the notion of bacteriophage-mediated integration of the *gtrIC* cluster into a pre-existing serotype.

**Conclusions:**

This is the first study to show that serotype 1c isolates from different geographical origins share an identical pattern of genetic arrangement, suggesting that serotype 1c strains may have originated from a single parental strain. Analysis of the sequence around the *gtrIC* cluster revealed a new site for the integration of the serotype converting phages of *S. flexneri*. Understanding the origin of new pathogenic serotypes and the molecular basis of serotype conversion in *S. flexneri* would provide information for developing cross-reactive *Shigella* vaccines.

**Electronic supplementary material:**

The online version of this article (doi:10.1186/s12866-016-0746-z) contains supplementary material, which is available to authorized users.

## Background

The lipopolysaccharide (LPS) of shigellae is known to exhibit a high degree of antigenic diversity. This diversity arises primarily from differences in the structure and composition of the O-antigen. *S. flexneri* serotypes (with the exception of serotype 6) contain the same basic O-antigen backbone, namely a repeating tetrasaccharide unit made up of one N-acetylglucosamine residue (Glc*N*Ac) and three rhamnose residues (RhaI, RhaII and RhaIII). Currently, there are at least 15 established *S. flexneri* serotypes, including the newly designated 1c and 7b subtypes [1], all of which are capable of causing shigellosis. There are also a few more putative new serotypes which are yet to be considered for possible official classification [2, 3]. Each of these serotypes contains a specific LPS-O antigen that is responsible for its particular serotype characteristics.

**Fig. 1:**
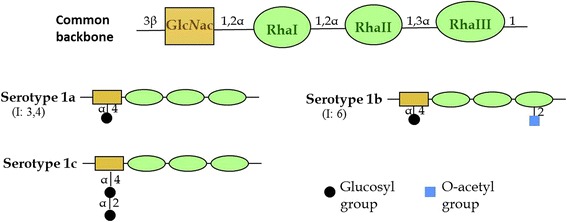
The chemical structure of the repeating tetrasaccharide units in the O-antigen of *S.flexneri* serotypes 1a, 1b and 1c

Serotype 1c, also known as 7a subtype of *S. flexneri*, emerged in the 1990s. The presentation of O-antigens in serotype 1c is unique, as it is the first example in which an α-D-Glcp-(1➔2)-α-D-Glcp-(termed kojibiosyl) group is added to the basic repeating unit of O-antigen [4]. Serotype 1c contains a disaccharide linked to the N-acetyleglucosamine in the basic tetrasaccharide repeating units, whereas serotype 1a and 1b strains contain only a single glucosyl group at the same site (Fig. 1).

The genetic mechanism responsible for O-antigen modification in serotype 1c was first elucidated by Stagg et al. [5]. The addition of the first glucosyl group is mediated by the previously characterised *gtrI* cluster found within a cryptic prophage at the *proA* locus in the bacterial chromosome. Transposon mutagenesis, performed to disrupt the gene responsible for the addition of the second glucosyl group, successfully identified the gene encoding the serotype 1c –specific O-antigen modification, which was designated as *gtrIC*. The *gtrIC* gene was present as part of a three gene cluster, arranged in a similar way to the *gtr* clusters present in other *S. flexneri* serotypes.

Adhikari et al. [6] earlier concluded that *gtrI* was integrated into *S. flexneri* by a bacteriophage via the tRNA^ThrW^*proA* site. Our preliminary analysis of the sequence adjacent to the *gtrIC* cluster suggested the possibility of another integration site for serotype 1c prophage [5]. We hypothesized that serotype 1c strains arose, following the introduction of the *gtrIC* gene cluster, via a second bacteriophage that got inserted into a separate location on the chromosome of an ancestral serotype 1a strain. In this study, we show that serotype 1c strains are genetically related through conserved *gtrIC* sequences, and that serotype 1c isolates share an identical pattern of genetic arrangement despite their different geographical origins. In addition, we report the identification of a new site for the integration of the serotype converting phages of the *S. flexneri* serotype 1c strain. The experiments and sequence analyses performed in this study provide further insights into the origin of this serotype.

## Methods

### Bacterial culturing conditions and media

The *S. flexneri* strains used in the study are listed in Table 1. Bacteria were grown aerobically (≅180-200 RPM) at 37 °C in Luria-Bertani (LB) broth or on LB agar supplemented with appropriate antibiotics. Unless stated otherwise, antibiotics (Sigma-Aldrich) were added at the following final concentrations: ampicillin (100 μg/mL); chloramphenicol (25 μg/mL); tetracycline (10 μg/mL); and kanamycin (50 μg/mL).

**Table 1:** Wild type *S. flexneri* strains used in this study

Strains	Derivation/other names	Reference	Description
SFL1416	NCTC #3	NCTC	Serotype 1a strain from NCTC (The National Collection of Type Cultures) London, UK
SFL1417	NCTC #5	NCTC	Serotype 1b strain from NCTC (The National Collection of Type Cultures) London, UK
Bangladesh			
SFL1492	K-480	[19]	Serotype 1b strain isolated in Bangladesh
SFL1493	K-647	[19]	Serotype 1a strain isolated in Bangladesh
SFL1496	K-218	[19]	Serotype 1b strain isolated in Bangladesh
SFL1499	K-143	[19]	Serotype 1b strain isolated in Bangladesh
SFL1501	K-265	[19]	Serotype 1c strain isolated in Bangladesh
SFL1504	K-212	[19]	Serotype 1c strain isolated in Bangladesh
SFL1613	Y394	[4]	Serotype 1c strain isolated in Bangladesh
Egypt			
SFL1683	ALX1592	[20]	Serotype 1c strain isolated in Egypt
SFL1684	ALX1592	[20]	Serotype 1c strain isolated in Egypt
SFL1685	ALX1869	[20]	Serotype 1c strain isolated in Egypt
SFL1686	ALX1929	[20]	Serotype 1c strain isolated in Egypt
SFL1687	ALX1930	[20]	Serotype 1c strain isolated in Egypt
SFL1688	ALX1938	[20]	Serotype 1c strain isolated in Egypt
SFL1689	ALX2463	[20]	Serotype 1c strain isolated in Egypt
SFL1690	ALX2539	[20]	Serotype 1c strain isolated in Egypt
SFL1691	ALX3216	[20]	Serotype 1c strain isolated in Egypt
SFL1692	ALX3326	[20]	Serotype 1c strain isolated in Egypt
Japan			
SFL1276	850325-1331	C. Sasakawa	Serotype 1b strain isolated in Japan
SFL1287	1649-17	C. Sasakawa	Serotype 1b strain isolated in Japan
SFL1288	1649-18	C. Sasakawa	Serotype 1a strain isolated in Japan
SFL1300	1649-30	C. Sasakawa	Serotype 1b strain isolated in Japan
Vietnam			
SFL1564	737	[21]	Serotype 1c (rough) strain isolated in Vietnam (Son Tay Province)
SFL1568	774	[21]	Serotype 1c (rough) strain isolated in Vietnam (Son Tay Province)
SFL1569	784	[21]	Serotype 1c strain isolated in Vietnam (Son Tay Province)
SFL1570	786	[21]	Serotype 1c (rough) strain isolated in Vietnam (Son Tay Province)
SFL1571	790	[21]	Serotype 1c strain isolated in Vietnam (Son Tay Province)
SFL1575	1216	[21]	Serotype 1c strain isolated in Vietnam (Son Tay Province)
SFL1576	1217	[21]	Serotype 1c (rough) strain isolated in Vietnam (Son Tay Province)
SFL1577	1233	[21]	Serotype 1c (rough) strain isolated in Vietnam (Son Tay Province)
SFL1578	1240	[21]	Serotype 1c strain isolated in Vietnam (Son Tay Province)
SFL1579	1244	[21]	Serotype 1c strain isolated in Vietnam (Son Tay Province)
SFL1580	1245	[21]	Serotype 1c strain isolated in Vietnam (Son Tay Province)
SFL1581	1247	[21]	Serotype 1c strain isolated in Vietnam (Son Tay Province)
SFL1582	1248	[21]	Serotype 1c strain isolated in Vietnam (Son Tay Province)
SFL1583	1249	[21]	Serotype 1c (rough) strain isolated in Vietnam (Son Tay Province)
SFL1584	1250	[21]	Serotype 1c (rough) strain isolated in Vietnam (Son Tay Province)
SFL1585	1251	[21]	Serotype 1c strain isolated in Vietnam (Son Tay Province)
SFL1586	1274	[21]	Serotype 1c strain isolated in Vietnam (Son Tay Province)
SFL1587	1292	[21]	Serotype 1c (rough) strain isolated in Vietnam (Son Tay Province)
SFL1588	1367	[21]	Serotype 1c strain isolated in Vietnam (Son Tay Province)
SFL1589	1387	[21]	Serotype 1c strain isolated in Vietnam (Son Tay Province)
SFL1590	1400	[21]	Serotype 1c strain isolated in Vietnam (Son Tay Province)
SFL1594	1432	[21]	Serotype 1c strain isolated in Vietnam (Son Tay Province)
SFL1595	1440	[21]	Serotype 1c (rough) strain isolated in Vietnam (Son Tay Province)
SFL1596	1447	[21]	Serotype 1c strain isolated in Vietnam (Son Tay Province)
SFL1597	1502	[21]	Serotype 1c strain isolated in Vietnam (Son Tay Province)
SFL1598	1587	[21]	Serotype 1c strain isolated in Vietnam (Son Tay Province)
SFL1600	1607	[21]	Serotype 1c strain isolated in Vietnam (Son Tay Province)
SFL1602	1670	[21]	Serotype 1c strain isolated in Vietnam (Son Tay Province)
SFL1603	1702	[21]	Serotype 1c strain isolated in Vietnam (Son Tay Province)
SFL1604	1711	[21]	Serotype 1c (rough) strain isolated in Vietnam (Son Tay Province)
SFL1605	1733	[21]	Serotype 1c strain isolated in Vietnam (Son Tay Province)
SFL1606	1804	[21]	Serotype 1c strain isolated in Vietnam (Son Tay Province)
SFL1607	1921	[21]	Serotype 1c strain isolated in Vietnam (Son Tay Province)
SFL1610	2238	[21]	Serotype 1c strain isolated in Vietnam (Son Tay Province)
SFL1556	NB1	[22]	Serotype 1c strain isolated in Vietnam (Nha Trang province)
SFL1557	NB2	[22]	Serotype 1c strain isolated in Vietnam (Nha Trang province)
SFL1558	NB7	[22]	Serotype 1c strain isolated in Vietnam (Nha Trang province)
SFL1561	NB545	[22]	Serotype 1c strain isolated in Vietnam (Nha Trang province)
SFL1562	NB644	[22]	Serotype 1c strain isolated in Vietnam (Nha Trang province)
SFL1565	747	[22]	Serotype 1c strain isolated in Vietnam (Nha Trang province)
SFL1566	749	[22]	Serotype 1c strain isolated in Vietnam (Nha Trang province)
SFL1567	752	[22]	Serotype 1c strain isolated in Vietnam (Nha Trang province)
SFL1572	1097	[22]	Serotype 1c strain isolated in Vietnam (Nha Trang province)
SFL1573	1127	[22]	Serotype 1c strain isolated in Vietnam (Nha Trang province)
SFL1599	1588	[22]	Serotype 1c strain isolated in Vietnam (Nha Trang province)
SFL1712	481NT2	[22]	Serotype 1c strain isolated in Vietnam (Nha Trang province)

### Serotyping

The serological features of the *S. flexneri* strains were determined by slide agglutination. A sterile loop was used to mix bacteria from LB agar plates with a drop of antibody on a glass slide. The slide was gently agitated while observing for agglutination. Negative controls were performed using 0.9 % NaCl instead of antibody. Isolates were tested using both commercially available monovalent antisera (Denka Seiken, Tokyo, Japan) and the monoclonal antibody reagent MASF Ic (Reagensia AB, Sweden) directed against type-specific somatic and group O factor antigens of *S. flexneri*.

### DNA techniques

Genomic DNA was isolated from an overnight culture using the Illustra™ bacteria Genomic Prep Mini Spin Kit (GE healthcare) in accordance with the manufacturer’s instructions. Oligonucleotide primers used for PCR were synthesized by Sigma-Aldrich (Australia), and are listed in Table 2. PCR was performed using PfuUltra II Fusion HS DNA Polymerase (Stratagene) in accordance with the manufacturer’s instructions. Purification of the PCR products was achieved using the Wizard SV Gel and PCR Clean Up system (Promega, Maddison, Wisconsin, USA). DNA sequencing was performed using the Big Dye Version 3.1 sequencing protocol, and was analysed with the ABI 3730 capillary sequence analyser at the Biomolecular Resources Facility, John Curtin School of Medical Research, Australian National University. Digestion of the DNA was performed using enzymes supplied by Fermentas.

**Table 2:** Primers used in this study

Primer name	Sequence	Description
GtrIc-F(BamHI)^a^	TTAGGATCCAGGGATTCAACTGATTGGC	Binds upstream of *gtrIC* (in *gtrB*_*IC*_).
GtrIcR2-BamHI	TGAGGATCCGACAGGATCAATCACCGC	Binds downstream of *gtrIC* stop codon.
DG_GtrA(Ic)F(SacI) ^b^	GTCGAGCTCTTGATGCTAAACTCTCACTTT	Binds to sequence 24–44 bp upstream of *gtrA*_*IC*_ start codon

^a^Primer pair of GtrIc-F(BamHI) and GtrIc-R2(BamHI), was optimal for the production of a single band which corresponded to the *gtrIC* gene

^b^Primer pair of DG_GtrA(Ic)F(SacI) and GtrIc-R2(BamHI) was the best choice for the *gtrIC* gene cluster

### Bioinformatics analysis

The DNA sequence was analysed for the presence of ORFs and *tRNA* genes using the open access software programmes myRAST (RASTserver.pm), CLC Main workbench 6.7 (CLCbio) and NCBI ORF finder, followed by manual inspection of the start codons and ribosome binding sequences of each ORF. Genes within ORFs were predicted based on homologies to known genes found by BLASTn and BLASTp searches, as well as by the presence of Shine-Dalgarno ribosome binding sites. The corresponding proteins were compared with the non-redundant protein database using the BLASTp and BLASTx programmes available from the National Centre for Biotechnology Information (http://www.ncbi.nlm.nih.gov). The protein level alignments were performed using CLUSTAL W [7] and BioEdit Sequence Alignment Editor [8].

### Southern blotting

Genomic DNA digestion was achieved by using DNA (1000 ng) in a total volume of 100 μl overnight digestion, with an appropriate restriction enzyme. Following an agarose gel electrophoresis of the digested genomic DNA samples, the DNA was transferred to a Hybond N+ nucleic acid transfer membrane (Amersham Biosciences) through capillary action. A *DIG* High Prime DNA Labelling and Detection Kit (Roche) was used to generate Digoxigenin (*DIG*) – labelled DNA probe. Hybridization of the membrane as well as detection were performed according to the kit manufacturer’s instructions. The membrane was viewed under a Fusion Chemiluminescence Camera (Fisher Biotech).

## Results and discussion

### Serotype 1c strains have a conserved *gtrIC* sequence

**Fig. 2:**
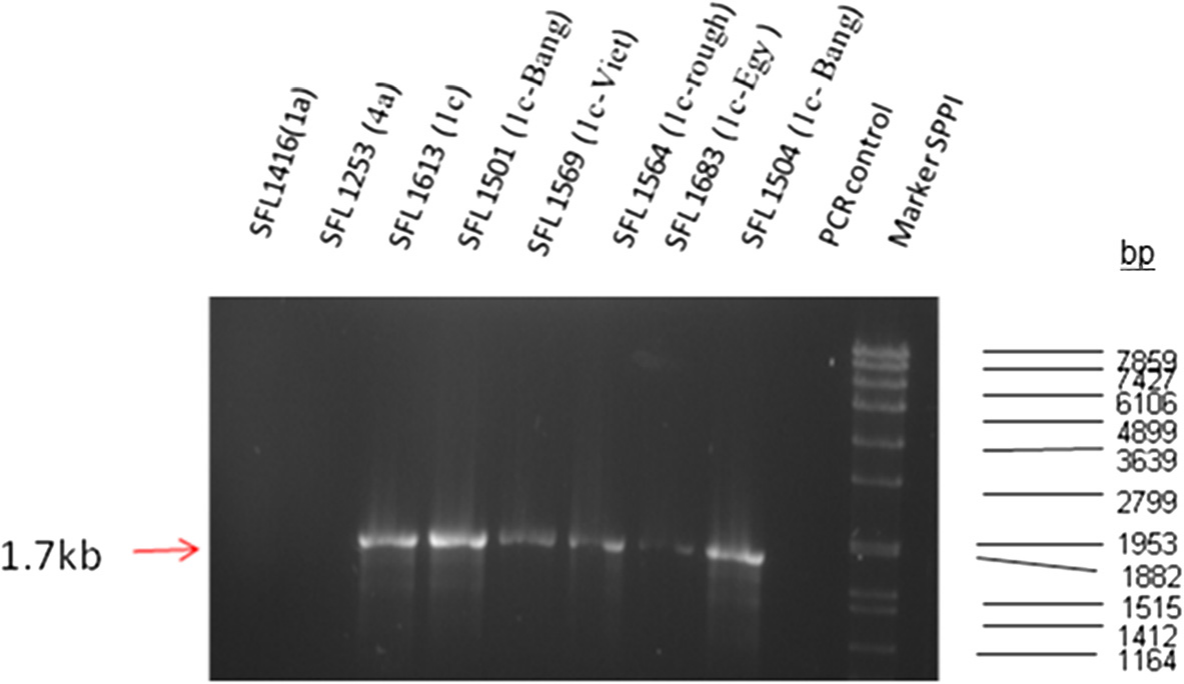
Detection of serotype 1c strains among a variety of *S. flexneri* strains using PCR amplification with the *gtrIC* specific primer pair. Amplification of *gtrIC* gene cluster product was visualised under UV light following agarose gel electrophoresis in the presence of ethidium bromide. Lane:1. SFL1416, serotype 1a; 2. SFL1253, serotype 4a; 3. SFL1613, serotype 1c strain isolated from Bangladesh; 4. SFL1501, serotype 1c strain isolated from Bangladesh; 5. SFL1569, serotype 1c strain isolated from Vietnam; 6. SFL1564, rough strain isolated from Vietnam; 7. SFL1683, serotype 1c strain isolated from Egypt;8. SFL1504, serotype 1c strain isolated from Bangladesh; 9. H_2_O control. 10. Expected sizes of PCR products are indicated by a red arrow, which was estimated using the DNA marker, SPPI

Until now, very little has been known about the extent of *gtrIC* conservation among *S. flexneri* 1c strains from different regions of the world. Therefore, in order to study the *gtrIC* homology and the prevalence of the putative *gtrIC* variants in various 1c isolates of patients from different ethnic and geographic origins, PCR was employed to detect the presence of the *gtrIC* gene. This was done concurrently with conventional agglutination tests. All strains which had positive serotype 1c agglutination results also produced a PCR amplicon of 1769 nt, corresponding to the presence of the *gtrIC* gene. As shown in Fig. 2, a PCR product of the same size was also produced in a rough serotype 1c strain which did not express serotype 1c specific O-antigen, and which therefore could not be typed by antisera. Furthermore, sequencing of the PCR amplicon in which the whole *gtrIC* cluster was amplified by primer pair of DG_GtrA(Ic)F(SacI) and GtrIc-R2(BamHI), revealed that the *gtrA*_*Ic*_ and *gtrB*_*Ic*_ genes from all the representative strains were exactly identical to each other. The results revealed that the serotype 1c strains had 100 % identical *gtrIC* gene nucleotide sequences as well as 100 % nucleotide identity for the whole *gtrIC* clusters (*gtrA*_*Ic*_*, gtrB*_*Ic*_ and *gtrIC* genes). This means that extreme conserved nucleotide sequences exist not only in the *gtrIC* locus, but also in the whole *gtrIC* cluster.

**Fig. 3:**
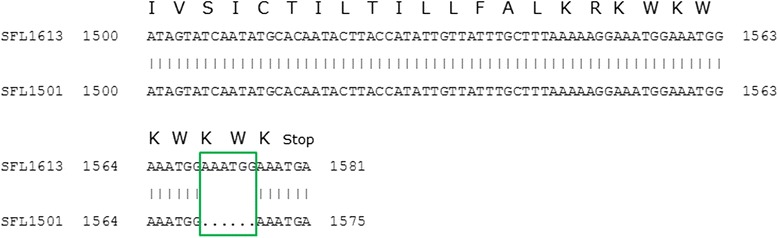
The comparison of the 3′ end of *gtrIC* sequence of SFL1501 to the published *gtrIC* sequence of SFL1613. The repeating GAAATG feature in both sequences. TGA depicts a stop codon

The only exception to the above was SFL1501, which contained the *gtrIC* gene with a 6-bp deletion (GAAATG). Interestingly, this deletion was one of four GAAATG repeats present at the 3′ terminus of *gtrIC* gene (Fig. 3). Perhaps the absence of one of the four repeats of tryptophan-lysine residues at the C-terminus does not affect the overall function of the GtrIc. It is possible that sequence redundancy and the repeated sequences compensate for this loss.

Based on the fact that a conserved nucleotide sequence exists and no silent mutation was detected in *gtrIC* and its cluster sequence, we speculate that Type 1c modification plays a vital role within *S. flexneri*, and may assist the bacteria to a certain extent in the invasion of the epithelial cells of the host organism.

### Serotype 1c isolates share an identical pattern of genetic arrangement despite differing geographical origins

Southern blotting with the *gtrIC* probe was used to reveal the upstream and downstream organization and distribution of the *gtrIC* gene cluster in different strains of *S. flexneri* serotype 1c. If the upstream and downstream organization of the *gtrIC* gene cluster are the same, two fragments should be expected with *Eco*32I digestion and one fragment for *Bam*HI digestion. If, on the other hand, there are any differences between the organization of the upstream and downstream regions, fragments of variable sizes should be produced. These data should not only cast light on the organization of the upstream and downstream of *gtrIC* gene clusters in different strains, but also allow the determination of the number of copies of the *gtrIC* locus present in the genome of various 1c isolates.

**Fig. 4:**
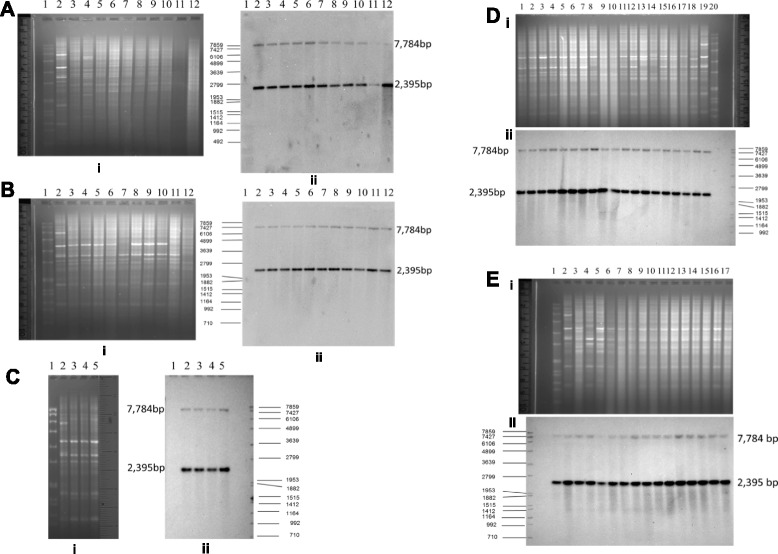
Southern Blot of *Eco*32I digested chromosomal DNA with a *gtrIC* probe. i. Agarose gel of digested genomic DNA. ii. Southern blot analysis of digested genomic DNA. **a** Egyptian serotype 1c strains. Lane 1. Marker SPP-I/*Eco*RI; 2. SFL1613 (control strain); 3. SFL1683; 4. SFL1684; 5. SFL1686; 6.SFL1687; 7. SFL1688; 8.SFL1689; 9. SFL1690; 10.SFL1691; 11.SFL1692; 12. SFL1685. **b** Bangladeshi serotype 1c strains. Lane 1. Marker SPP-I/*Eco*RI; 2. SFL1613 (control strain); 3. SFL1500; 4. SFL1502; 5. SFL1503. **c** Vietnamese (Son Tay Province) serotype 1c strains. Lane 1. SPP-I/*Eco*RI; 2. SFL1613 (control strain); 3. SFL1564; 4. SFL1568; 5. SFL1569; 6.SFL1571; 7. SFL1575; 8.SFL1576; 9. SFL1577; 10.SFL1578; 11.SFL1579; 12. SFL1570. **d** Vietnamese Serotype 1c strains - Son Tay Province. (Isenbarger et al. 2001). Lane 1. SFL1580; 2. SFL1581; 3. SFL1582; 4. SFL1583; 5. SFL1584; 6.SFL1585; 7. SFL1586; 8.SFL1587; 9. SFL1588; 10.SFL1589; 11.SFL1590; 12. SFL1594.; 13.SFL1596; 14. SFL1597. 15.SFL1598; 16. SFL1600; 17.SFL1602; 18. SFL1603;19. SFL1613 (positive control strain) 20. Marker SPP-I/*Eco*RI. **e** Nha Trang, Vietnam (5 from Isenbarger et al. 2001, 13 from Prof. Cam PD). Lane 1. Marker SPP-I/*Eco*RI; 2. SFL1604; 3. SFL1605; 4. SFL1606; 5. SFL1607; 6.SFL1610; 7. SFL1556; 8.SFL1557; 9. SFL1558; 10.SFL1561; 11.SFL1562; 12. SFL1565.; 13.SFL1566; 14. SFL1567. 15.SFL1572; 16. SFL1573; 17.SFL1599; 18. SFL1712;19. SFL1613 (positive control strain) 20. Marker SPP-I/*Xba*I

A total of sixty-nine different serotype 1c isolates, obtained from Bangladesh, Egypt and Vietnam, were screened. The *Eco*321-digested genomic DNA of all the serotype 1c strains, when probed with *gtrIC*, showed two bands: a 7784 bp and a 2395 bp fragment. This was the same as the positive control SFL1613 (Fig. 4). No bands were present in the negative control.

*Bam*HI-digested genomic DNA was used to examine the genetic arrangement of the downstream region of *gtrIC*. In all the serotype 1c strains evaluated (one Bangladeshi, four Egyptian, four Vietnamese from Son Tay province, and four Vietnamese from NhaTrang province), one band corresponding to the 12,500 bp fragment was observed when probed with *gtrIC* (Additional file 1: Figure S1).

The findings from both sets of Southern blot analysis show that all the serotype 1c strains had the same genetic organization upstream and downstream of the *gtrIC* cluster, despite their different geographic origins; also, that they were flanked by the same insertion sequences and located next to the *yejO* locus (Additional file 2: Figure S2). As the serotype 1c strains used in this Southern hybridization study were obtained from several different geographic locations, it would have been reasonable to expect that these *S. flexneri* isolates would have different structures of the *gtrIC* cluster. Moreover, some might well have contained an intact bacteriophage or prophage sequence, which would have resulted in different genetic arrangements of the sequence surrounding the *gtrIC* gene. However, our findings surprisingly showed the organization of the *gtrIC* gene cluster to be universal and conserved in all the *S. flexneri* serotype 1c strains examined. The Southern hybridization results also revealed that only one copy of *gtrIC* was present in all of the tested strains – which suggests that all the serotype 1c strains are likely to have originated from a single clone.

Findings like ours are not unique. Similar findings were also reported in *Streptococcus pneumoniae*, with different types of 37 clinical isolates from two different continents (Europe and America) having an identical *tts* gene directing the formation of type 37 capsular polysaccharide [9]. These isolates too constituted a highly related strain cluster (clonal complex), suggesting that every type 37 pneumococcus found globally had originated from a single parental clone.

In the same vein, a study conducted by Frosch et al. [10] using Southern blot analysis revealed a strong homology between the functional regions of the *cps* locus of different meningococcal serogroups. A further study by Frosch et al. [11] showed the molecular organization of the capsule gene (*cps*) loci in different serogroups of *Neisseria meningitidis* to be very similar to that of *E. coli* and *Haemophilus influenzae.* These authors concluded that the strongly homologous organization of the capsule gene loci in *N. meningitidis*, *E. coli* and *H. influenzae* point to a common evolutionary origin of capsule production in Gram-negative bacteria expressing group II capsular polysaccharides.

### Origin of the *gtrIC* modification in *S. flexneri* serotype 1c strains

The *gtrIC* modification we observed may have originated either through a serotype 1a strain gaining the *gtrIC* or through a serotype 1c strain losing the *gtrIC* function. If a serotype 1a strain was derived from an ancestral serotype 1c strain, due to the *gtrIC* cluster in the serotype 1a strain having been disrupted by either insertion elements or through gene deletion, then remnant(s) of the *gtrIC* gene or the gene cluster would exist in the genome of serotype 1a strains (Additional file 3: Figure S3).

**Fig. 5:**
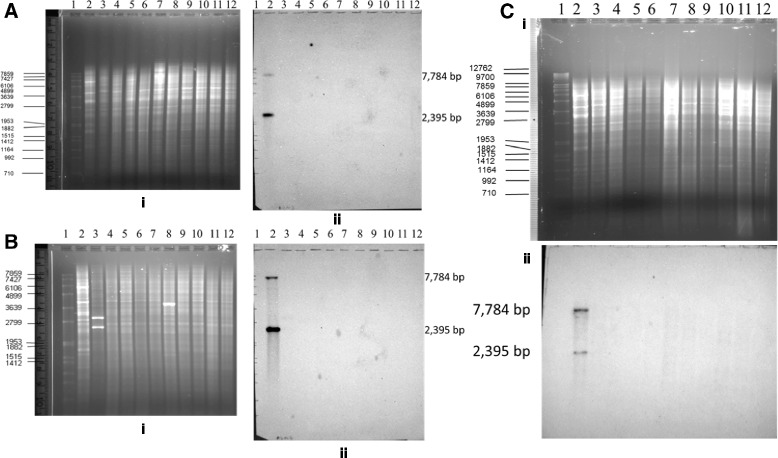
Southern Blot of *Eco*32I-digested chromosomal DNA. i. Agarose gel of digested genomic DNA. ii. Southern blot analysis of digested genomic DNA. **a** Bangladeshi, and Japanese serotypes 1a and 1b strains, with a *gtrIC* probe. Lane 1. Marker SPP-I/*Eco*RI; 2. SFL1613 (control strain); 3. SFL1287 (1a-Jpn); 4. SFL1288 (1a-Jpn); 5. SFL1492 (1a-Bangladesh); 6.SFL1493(1a-Bangladesh); 7. SFL1494(1a-Bangladesh); 8.SFL1495(1a-Bangladesh); 9. SFL1496(1b-Bangladesh); 10.SFL1497(1b-Bangladesh); 11.SFL1498(1b-Bangladesh); 12. SFL1499(1b-Bangladesh). **b** UK and Japanese serotype 1b strains, with a *gtrIC* probe. Lane 1. Marker SPP-I/*Eco*RI; 2. SFL1613 (control strain); 3. B1118 (control negative strain); 4. SFL1417 (1b-NCTC-UK); 5. SFL276 (1b-Japan); 6.SFL1289(1b-Japan); 7. SFL1300(1b-Japan); 8.SFL1309(1b-Japan); 9. SFL1315(1b-Japan); 10.SFL1316(1b-Japan); 11.SFL1277(1b-rough-Japan); 12. SFL1278(1b-rough-Japan). **c** Ten selected UK, Bangladeshi and Japanese serotype 1b strains, with a *gtrIC* cluster probe. Lane 1. SPP-I/*Eco*RI; 2. SFL1613 (control strain); 3. SFL1287(1a-Japan); 4. SFL1300 (1b-NCTC-UK); 5. SFL1315 (1b-Japan); 6.SFL1316(1b-Japan); 7. SFL1499(1b-B); 8.SFL1498(1b-Rough); 9. SFL1497(1b-B); 10.SFL1496(1b-B); 11.SFL1417(1b-UK); 12. SFL1277(1b-rough-Japan)

In order to investigate if remnants of the *gtrIC* gene or gene cluster exist in serotype 1a strains, a Southern blot analysis was performed using the *gtrIC* and *gtrIC* cluster probes. Serotype 1b strains were also included in this analysis because they have the same α1➔4 linkage to N-acetylGlc as serotype 1a strains. All together six serotype 1a and thirteen serotype 1b strains, isolated from Bangladesh, the UK and Japan, were analysed with Southern blotting. The genomic DNAs from these strains were digested with *Eco*32I and probed with DIG-labelled *gtrIC*. None of the screened serotype 1a or serotype 1b strains showed a detectable *gtrIC* gene remnant (Fig. 5a and b).

Additional Southern blotting with *gtrIC* cluster as a probe was then performed to confirm the results obtained. We thought that the *gtrIC* cluster (containing the *gtrA*_*Ic*_, *gtrB*_*Ic*_ and *gtrIC* genes as an operon) would be able to act as a more comprehensive probe to detect remnants of *gtrA*_*Ic*_, *gtrB*_*Ic*_ and *gtrIC*. Ten of the previously screened 1a and 1b strains were selected for this additional assay. Other than the control SFL1613 strain, which showed two bands of 2395 and 7784 bp as expected, the rest of the samples did not produce any significant band (Fig. 5c). This clearly confirmed that no remnant of *gtrA*_*Ic*_, *gtrB*_*Ic*_ or *gtrIC* existed in any of the screened serotype 1a and 1b strains.

The lack of the *gtrIC* gene specifically, and more broadly of the *gtrIC* gene cluster, from the genomic DNA of the serotype 1a and 1b strains indicates that the *gtrIC* cluster did not exist in an ancestor of the serotype 1a or 1b strains. This finding thus rules out the “loss of *gtrIC* function” hypothesis, and proves that serotype 1a/1b strains did not derive from a serotype 1c strain. The more likely explanation, therefore, is that the *gtrIC* cluster was inserted into an *S. flexneri* serotype 1a strain via a bacteriophage. This hypothesis is consistent with the findings from the analysis of the sequence surrounding the *gtrIC* and *gtrI* clusters in serotype 1c strains.

### Presence of *EPEC* gene in *S. flexneri* serotype 1c strains and sequences surrounding *gtrIC* cluster

**Fig. 6:**
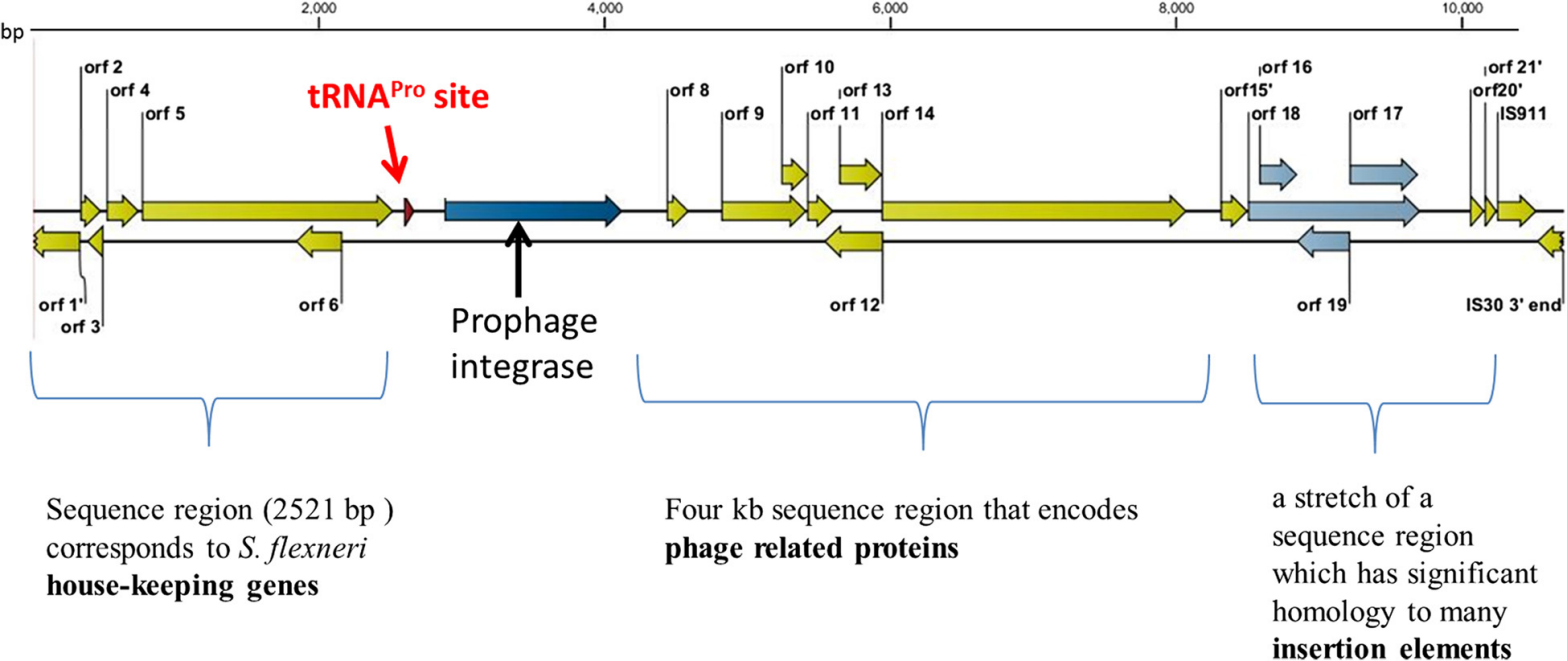
Linear representation of the 10,243 bp nucleotide sequence further upstream from the previously sequenced *gtrIC* cluster in SFL1613. The first line shows the nucleotide sequence scale in base pairs. The second and third lines show the distribution of all ORFs, with horizontal arrows denoting the direction of transcription. Dark blue represents ORF in the prophage integrase region, light blue represents ORFs in the insertion sequence region

We had previously studied a 7241 bp upstream and 11,906 bp downstream region surrounding the *gtrIC* cluster in SFL1613 and shown this to contain the IS629 isoform, ISEhe3 fragment, hypothetical ORF proteins and several housekeeping genes (*yejO, narP, ccmH, dsbE, ccm*) at the 3′ end, as well as insertion elements (IS911 interrupted three times by IS30, a group II intron and a putative transposase) at the 5′end [5]. A continuous 4700 bp sequence of the 7241 bp nucleotide upstream was 98 % identical to a region in the pB171 plasmid of the Enteropathogenic *E. coli*. Therefore, in order to identify whether SFL1613 contained any further sequence in common with pB171, additional sequencing (further upstream of the previously published 7.2 kb sequence) was performed. A stretch of the 10,243 bp nucleotide sequence further upstream of the *gtrIC* gene cluster was obtained by primer walking. A bioinformatics analysis of the 10,243 bp sequence revealed 21 putative open reading frames (ORFs); of which 17 were complete and 4 incomplete. Of the 17 complete ORFs, 16 were predicted to encode proteins which were significantly homologous with known proteins, while 1 ORF had no region of significant homology with proteins in the current database (Fig. 6 and Table 3).

**Table 3:** Sequence analysis of the 10243 bp fragment further upstream of *gtrIC* cluster

ORF (gene name) or feature	Nt position^a^	Gene size (bp)	No of amino acids encoded	Database search results				
				Feature or protein (Saiz, aa)	Source (accession no.)	Identity (%)	Positive (%)	BlastP *E* value
*orf 1’ (ndpA* or *yejK)*	Complement (1..332)	332 of 1007	111	Nucleoid-associated protein ndpA (335aa)	*Shigella flexneri* K-272 (EGK22467.1)	110/110 (100 %)	110/110 (100 %)	7e-72
				DNA-associated protein (335aa)	*Escherichia coli* BL21(DE3)(YP_002999856.1)	110/110 (100 %)	110/110 (100 %)	8e-72
				Nucleoid-associated protein NdpA (335aa)	*Shigella boydii* Sb227 (YP_408543.1)	110/110 (100 %)	110/110 (100 %)	8e-72
					*Shigella boydii* CDC 3083-94			
				Nucleoid-associated protein YejK (335aa)	(YP_001879481.1)	110/110 (100 %)	110/110 (100 %)	8e-72
*orf 2*	331..477	147	48	Hypothetical prot. HMPREF9346_02485, (48aa)	*Escherichia coli* MS 119–7 (ZP_07102777.1)	48/48 (100 %)	48/48 (100 %)	7e-26
				Hypothetical prot. HMPREF9552_01955, (48aa)	*Escherichia coli* MS 198-1(ZP_07116146.1)	48/48 (100 %)	48/48 (100 %)	7e-26
				Hypothetical prot. HMPREF9547_02658, (48aa)	*Escherichia coli* MS 175–1 (ZP_07169115.1)	48/48 (100 %)	48/48 (100 %)	7e-26
				Putative ABC transporter permease protein,(303aa)	*Streptomyces roseosporus*	13/25 (52 %)	17/25 (68 %)	8.6
					NRRL11379(ZP_04709202.1)			
				ABC transporter permease protein, (303aa)	*Streptomyces roseosporus* NRRL	13/25 (52 %)	17/25 (68 %)	8.6
					15998(ZP_06584909.1)			
				ABC transporter permease protein (303aa)	*Streptomyces roseosporus* NRRL	13/25 (52 %)	17/25 (68 %)	8.6
					15998 (EFE75370.1)			
*orf 3*	Complement (377..493)	117	38	Hypothetical prot. ECSTEC7V_2603, (54aa)	*Escherichia coli* STEC_7v (EGE64095.1)	38/38 (100 %)	38/38 (100 %)	4e-19
				hypothetical prot. EcE24377A_2485, (39aa)	*Escherichia coli* E24377A (YP_001463540.1)	38/38 (100 %)	38/38 (100 %)	9e-19
				Hypothetical protein EcHS_A2325, (39aa)	*Escherichia coli* HS (YP_001458987.1)	38/38 (100 %)	38/38 (100 %)	9e-19
				Hypothetical protein SbBS512_E077(39aa)	*Shigella boydii* CDC 3083–94 (YP_001879480.1)	38/38 (100 %)	38/38 (100 %)	9e-19
*orf 4 (yejL)*	514..741	228	75	*yejL* gene product (75aa)	*Shigella flexneri* 2a str. 301(NP_708086.1)	75/75 (100 %)	75/75 (100 %)	5e-46
				Hypothetical protein S2403(75aa)	*Shigella flexneri* 2a str. 2457 T (NP_837801.1)			
				Hypothetical protein SFV_2265 (75aa)	*Shigella flexneri* 5 str. 8401 (YP_689686.1)			
*orf 5 (yejM)*	761..2521	1761	586 (2 domains detected)	*yejM* gene product (586aa)	*Shigella flexneri* 2a str. 301(NP_708087.1)	586/586 (100 %)	586/586 (100 %)	0.0
				Sulfatase (586aa)	*Shigella flexneri* 2a str. 2457 T (NP_837802.1)			
				*yejM* gene product (586aa)	*Shigella flexneri* 2002017 (YP_005727919.1)			
*tRNA-Pro*	2596..2669	74	NA	*tRNA-Pro* (74 bp)	*Escherichia coli* str. K-12 substr. W3110 (NC_007779.1)	74/74 (100 %)^c^	NA	1e-30^c^
*orf 6*	Complement (1838..2164)	327	108	No significant homology				
*orf 7 (int)*	2879..4123	1245	414	Prophage integrase (413aa)	*Shigella boydii* CDC 3083–94 (YP_001879477.1)	412/412 (100 %)	412/412 (100 %)	0.0
				Prophage CP4-57 integrase (414aa)	*Escherichia coli* TX1999 (EGX23085.1)	408/414 (99 %)	410/414 (99 %)	0.0
				integrase (414aa)	*Escherichia coli* 042 (YP_006096729.1)	403/414 (97 %)	409/414 (99 %)	0.0
*orf 8*	4398..4589	192	63	Putative prophage regulatory protein (63aa)	*Escherichia coli* 042 (YP_006096730.1)	59/63 (94 %)	63/63 (100 %)	4e-35
				Hypothetical prot. SbBS512_E0760 (51aa)	*Shigella boydii* CDC 3083–94 (YP_001879474.1)	49/51 (96 %)	51/51 (100 %)	2e-27
				Transcriptional regulator, AlpA family (68aa)	*Escherichia coli* 53638 (ZP_03002982.1)	43/61 (70 %)	52/61 (85 %)	2e-24
				CP4-57 regulatory protein (AlpA) family protein (68aa)	*Escherichia coli* UMNF18 (AEJ57337.1)	42/61 (69 %)	52/61 (85 %)	3e-24
*orf 9*	4816..5412	597	198 *[F + 1]*	Putative prophage protein (198aa)	*Escherichia coli* 042 (YP_006096731.1)	165/198 (83 %)	171/198 (86 %)	4e-114
				Putative prophage protein (198aa)	*Escherichia coli* DEC7A (EHV77794.1)	151/198 (76 %)	163/198 (82 %)	7e-100
				Immunity region (569aa)	*Escherichia coli* STEC_94C	78/153 (51 %)	96/153 (63 %)	2e-34
*orf 10*	5235..5423	189	62 *[F + 3]*	Hypothetical prot. SbBS512_E0759 (62aa)	*Shigella boydii* CDC 3083–94 (YP_001879473.1)	62/62 (100 %)	62/62 (100 %)	1e-37
				Hypothetical protein SFK315_2596 (62aa)	*Shigella flexneri* K-315 (EIQ20710.1)	61/62 (98 %)	61/62 (98 %)	4e-36
				Conserved hypothetical protein (62aa)	*Escherichia albertii* TW07627 (ZP_02901801.1)	50/62 (81 %)	53/62 (85 %)	3e-29
*orf 11*	5416..5601	186	61	Hypothetical prot. SbBS512_E0758 (61aa)	*Shigella boydii* CDC 3083–94 (YP_001879472.1)	61/61 (100 %)	61/61 (100 %)	3e-35
				Putative prophage protein (61aa)	*Escherichia coli* 042 (YP_006096732.1)	60/61 (98 %)	60/61 (98 %)	5e-34
*orf 12*	complement (5536..5949)	414	137	Hypothetical prot. ECe0006 (95aa)	*Escherichia coli* (ABM53624.1)	43/92 (47 %)	54/92 (59 %)	6e-18
				Hypothetical prot. c1494(95aa)	*Escherichia coli* CFT073 (NP_753403.1)	44/93 (47 %)	55/93 (59 %)	9e-18
				Hypothetical prot. SBO_2130 (95aa)	*Shigella boydii* Sb227 (YP_408537.1)	42/92 (46 %)	53/92 (58 %)	2e-17
*orf 13*	5641..5940	300	99	Hypothetical prot. SbBS512_E0757 (99aa)	Shigella boydii CDC 3083–94 (YP_001879471.1)	99/99 (100 %)	99/99 (100 %)	2e-67
				Hypothetical prot. EcoM_00008 (99aa)	*Escherichia coli* WV_060327(EFW72294.1)	76/99 (77 %)	82/99 (83 %)	1e-47
				Hypothetical bacteriophage prot.(99aa)	*Escherichia coli* H299 (ZP_08382870.1)	75/99 (76 %)	81/99 (82 %)	6e-47
				Hypothetical bacteriophage prot.(99aa)	*Shigella dysenteriae* 1012 (ZP_03066472.1)	72/99 (73 %)	79/99 (80 %)	1e-44
				Bacteriophage protein (99aa)	*Shigella flexneri* 2a str. 301(NP_707045.1)	67/99 (68 %)	78/99 (79 %)	1e-41
*orf 14*	5937..8072 bacteriophage P4-DNA primease	2136	711	Hypothetical prot. SbBS512_E0756 (711aa)	*Shigella boydii* CDC 3083–94 (YP_001879470.1)	711/711 (100 %)	711/711 (100 %)	0.0
				Hypothetical prot. SFK315_2598 (711aa)	*Shigella flexneri* K-315 (EIQ20712.1)	674/709 (95 %)	691/709 (97 %)	0.0
				Putative prophage protein(712aa)	*Escherichia coli* 042 (YP_006096734.1)	630/712 (88 %)	658/712 (92 %)	0.0
				Putative prophage DNA primase (711aa)	*Escherichia coli* DEC7A,C,D,E, EPECa12(EHV77797.1,86415.1, 91550.1, EHW01270.1 EIQ62754.1)	621/711 (87 %)	652/711 (92 %)	0.0
				DNA Primease, phage-associated (713aa)	*Escherichia coli* PA5 (ZP_02787536.1)	563/713 (79 %)	618/713 (87 %)	0.0
				Putative prophage primase (693aa)	*Escherichia coli* 042 (YP_006096469.1)	520/693 (75 %)	579/693 (84 %)	0.0
*orf 15’*	8309..8500	192	64	Putative single stranded DNA-binding protein (141aa)	*Shigella boydii* CDC 3083–94 (YP_001879469.1)	64/64 (100 %)	64/64 (100 %)	3e-38
				Putative single stranded DNA-binding protein of prophage (136aa)	*Escherichia coli* IAI39 (YP_002407987.1)	59/64 (92 %)	62/64 (97 %)	9e-35
				Putative single-strand DNA binding prophage protein (141aa)	*Escherichia coli* 042 (ref|YP_006096735.1)	60/64 (94 %)	62/64 (97 %)	1e-34
*orf 16*	8580..8846	267	88	transposase (88aa)	*Escherichia coli* CFT073 (NP_754364.1)	86/88 (98 %)	88/88 (100 %)	2e-55
				transposase (88aa)	*Escherichia coli* UTI89 (YP_541223.1)	86/88 (98 %)	86/88 (98 %)	86/88 (98 %)
				IS1400 transposase A (88aa)	*Escherichia coli* 536 (YP_669883.1)			
				trp1400A gene product (95aa)	*Erwinia billingiae* Eb661(YP_003743077.1)	81/88 (92 %)	87/88 (99 %)	3e-52
				IS1400 transposase A (95aa)	*Yersinia enterocolitica* subsp. enterocolitica 8081 (YP_001004058.1)	80/88 (91 %)	86/88 (98 %)	6e-52
*orf 17*	9212..9691	480	159	Insertion element IS407 family protein(159aa)	*Escherichia coli* MS 107–1 (ZP_07096798.1)	151/159 (95 %)	156/159 (98 %)	8e-109
				transposase B (182aa)	*Edwardsiella ictaluri* 93–146 (YP_002932372.1)	151/159 (95 %)	152/159 (96 %)	7e-108
				Integrase core domain-containing protein (236aa)	*Escherichia fergusonii* B253 (EGC09228.1)	151/159 (95 %)	156/159 (98 %)	2e-107
				InsK (207aa)	*Salmonella enterica* subsp. enterica serovar Montevideo str. SARB31 (EHL38295.1)	150/159 (94 %)	155/159 (97 %)	1e-106
				Transposase B (233aa)	*Salmonella enterica* subsp. enterica serovar Kentucky str. CDC 191 (ZP_03224077.1)	151/159 (95 %)	156/159 (98 %)	2e-106
				IS1400 transposase B (159aa)	*Escherichia coli* 536 (YP_669884.1)	148/159 (93 %)	152/159 (96 %)	2e-106
*orf 18*	8580..9691	1112	370	Putative transposase (370aa)	*Salmonella enterica* subsp. VII (CAX68025.1)	351/370 (95 %)	358/370 (97 %)	0.0
				Transposase (370aa)	*Salmonella enterica* subsp. enterica serovar Enteritidis str. P125109 (YP_002244693.1)	350/370 (95 %)	352/370 (95 %)	0.0
				Transposase (370aa)	*Salmonella enterica* subsp. enterica serovar Gallinarum str. 287/91 (YP_002227530.1)	349/370 (94 %)	349/370 (94 %)	0.0
*Orf19*	Complement (8843..9217)	375	122	Hypothetical prot. HMPREF9345_01631(122aa)	*Escherichia coli* MS 107–1 (ZP_07096799.1)	108/122 (89 %)	115/122 (94 %)	1e-74
				Hypothetical protein UUU_27350 (124aa)	*Klebsiella pneumoniae* subsp. pneumoniae DSM 30104 (EJK89616.1)	100/124 (81 %)	111/124 (90 %)	5e-68
*orf 20’*	10055..10156	102	34	Transposase family protein (63aa)	*Shigella flexneri* J1713 (gb|EGM62085.1)	34/34 (100 %)	34/34 (100 %)	1e-15
				Transposase IS3/IS911 (40aa)	*Shigella flexneri* K-218 (EGK22768.1)	34/34 (100 %)	34/34 (100 %)	1e-15
				ISEhe3 orfA (71aa)	*Shigella sonnei* 53G (YP_005457329.1)	34/34 (100 %)	34/34 (100 %)	1e-15
				ISEhe3 orfA (92aa)	*Shigella flexneri* 5 str. 8401 (YP_688117.1)	34/34 (100 %)	34/34 (100 %)	2e-15
				ISEhe3 orfA (92aa)	*Shigella flexneri* 2a str. 2457 T (NP_836231.1)	34/34 (100 %)	34/34 (100 %)	2e-15
*orf 21’*	10157..10243	87	NA	Insertion sequence IS911 (1250 bp)^c^	*Shigella dysenteriae* (X17613.1)	86/87 (99 %)^c^	NA	2e-35^c^

^a^The position relative to the 10,244 bp fragment is indicated

^b^NA, not applicable

’Partial open reading frame

^c^On the basis of nucleotide sequence homology, percentage and E-value of BlastN database search

*Note*: all nt positions include the stop codon, while the aa length does not include the stop codon

2521 bp of sequence at the beginning of the 10,243 bp sequence is a section of a sequence region which corresponds to the *S. flexneri* house-keeping genes *yejK* (*orf 1’*), *yejL* (*orf 4*), *yejM* (*orf 5*), and to hypothetical proteins (*orf 2, 3& 6*) (Fig. 6 and Table 3). Further sequence analysis found this stretch of the SFL1613 sequence to be >99 % identical to that found in the *S. flexneri* serotype 2a strain 2457 T. A BlastP search revealed that the protein encoded by *orf 6* has no significant homology to any existing protein in the database. Meanwhile, the protein encoded by *orf-7* exhibited a high level of homology (E-value of 0.0) and 100 % (413/413aa) identity with a prophage integrase of *Shigella boydii* CDC3083-94 (NC_010658.1) (Table 3). Interestingly, the tRNA^Pro^ which was not identified in the previously published 19.1 kb fragment [5] was identified in this extended 10.2 kb fragment. It is located 210 bp upstream of the prophage integrase (Fig. 6).

It is also noteworthy that a tRNA^Pro^ gene, previously identified as being located between the *yejM* and *yejO* genes in *S. flexneri* serotype 2a (2457 T) and serotype 5a (8401) strains [12, 13], was found by this study to be located in the region upstream of the *gtrIC* cluster and adjacent to the *yejM*. These findings, together with the fact that prophage integrase and prophage related genes were located beside the tRNA^Pro^ gene, strongly suggest that the integration of a bacteriophage appears to have occurred in SFL1613, via the tRNA^Pro^site. The tRNA genes have previously been shown to be a common integration site for bacteriophage [14–16].

Four kb downstream of the prophage integrase and tRNA^Pro^ is a stretch of sequence code for *orf-8* to *orf-15’* proteins, whose functions are known to be associated with the bacteriophage lifestyle. This includes a truncated Xsingle stranded DNA-binding prophage protein, plus a few complete prophage hypothetical proteins such as a putative prophage regulatory protein, three putative prophage proteins, a bacteriophage DNA primase and prophage integrase, as annotated in the genome of *E. coli* 042 (gene bank accession number NC017626.1) [17]. This suggests that this stretch of sequence (upstream of the *gtrIC* cluser) was in fact derived from a phage.

Immediately upstream of the previously published 7241 bp sequence is a stretch of a sequence region which has significant homology to a number of insertion elements such as IS1400 (*orfs 16–18*), a hypothetical protein (*orf 19*), ISEhe3 (*orf 20’*), and IS911(*orf 21’*), all related to *Shigella spp, Salmonella spp and E. coli*. Conserved domains were detected in *orf-16* from the NCBI’s Conserved Domains Database*.* The highest scoring match was the HTH Hin-like domain, which is a family of DNA-binding domains unique to bacteria and represented by the Hin protein of *Salmonella*. The Hin recombinase induces the site-specific inversion of a chromosomal DNA segment containing a promoter, which controls the alternate expression of two genes by reversibly switching orientation. The rve_3 (pfam13683), integrase core domain, which mediates integration of a DNA copy of the viral genome into the host chromosome, was detected in *orf-17* [18].

Database searches and careful analysis of the 10,243 bp of nucleotide sequence and corresponding proteins in this region revealed no further sequence common to pB171 of EPEC.

The sequencing results of the 19.1 kb published sequence plus the extended 10.2 kb sequence (obtained from this study) clearly indicate that the organization of the *att* sites, *glucosyltransferase* (*gtrIC*) genes and *int* in the SFL1613 chromosome is reminiscent of a prophage, although it appears that more than half of the phage genome has been deleted. Our results also suggest that tRNA^Pro^ (upstream of the *gtrIC* gene cluster) and the *yejO* locus (downstream of *gtrIC* gene cluster) define the boundaries of the phage DNA in this area of the SFL1613 chromosome. A homology analysis of the proteins encoded by *orf 8* through *orf 15’* suggests that this region of the sequence is a prophage-related sequence. Furthermore, a Blast search matching with the enteroaggregative *E. coli* (EAEC) strain 042 database suggests that the 2 kb sequence downstream of the *gtrIC* cluster, located between the *yejO* locus and the IS*629*, is in fact derived from a phage [5]. These two findings clearly show that both the upstream and downstream of the *gtrIC* cluster are composed of prophage sequences which have been disrupted by various mobile genetic elements.

Another interesting observation to emerge from this study was the presence of at least 8 different insertion sequences in both the 19.1 kb and the extended 10.2 kb fragments (see Fig. 6 and Additional file 2: Figure S2). Given the large number of insertion sequences occurring in this region, it is reasonable to assume that the insertion of bacteriophage via the tRNA^Pro^ site (*attL*) was subsequently disrupted by insertion elements and consequently resulted in the deletion of the *attR* site of the tRNA^Pro^ in SFL1613.

## Conclusion

This study provides molecular insights into the novel *S. flexneri* serotype 1c strain, as well as the *gtrIC* gene cluster that drives its unique immune recognition. This is the first study to show that serotype 1c isolates share an identical pattern of genetic arrangement despite their differing geographic origins, suggesting that serotype 1c strains may have originated from a single parental strain. The gene cluster responsible for Type 1C modification appears to have emerged in the *S. flexneri* serotype 1a via a bacteriophage integrated into the tRNA^Pro^ locus.

These findings expand our knowledge of the Type 1C modification of *Shigella*, and shed light on the genetic distribution of the *gtrIC* locus in serotype 1c strains. This new information will be useful for future *Shigella* research, and particularly for the design of safe and effective multivalent or cross-reactive vaccines against shigellosis.

## Abbreviations

*DIG*,Digoxigenin; EAEC, enteroaggregative *E. coli*; Glc*N*Ac, N-acetylglucosamine residue; LPS, lipopolysaccharide; ORF, open reading frame; Rha, rhamnose.

## Additional files

Additional file 1: Figure S1. *Southern blot of *Bam*HI-digested genomic DNA probe with the *gtrIC* gene. (i) Agarose gel of digested genomic DNA. (ii) Southern blot analysis of digested genomic DNA. Lane 1. SPP-I/*Xba*I; 2. SFL1502; 3. SFL1684; 4. SFL1685; 5. SFL1686; 6.SFL1687; 7. SFL1575; 8.SFL1576; 9. SFL1578; 10.SFL1579; 11.SFL1556; 12. SFL1557.; 13.SFL1558; 14. SFL1712; 15.SFL1613; 16. Marker SPP-I/*Eco*RI. (DOCX 240 kb) Additional file 2: Figure S2. *The *gtrIC* cluster and surrounding 19,147 bp sequence in serotype 1c strain SFL1613 [5]. (DOCX 114 kb) Additional file 3: Figure S3. *Schematic diagram illustrating two hypotheses that could potentially explain the evolution of the serotype 1c strain. (A) The *gtrIC* insertion hypothesis. (B) The deletion of *gtrIC* hypothesis causing the loss of functional gtrIc modification. (DOCX 115 kb)
