# Supplementary figures and images for: Shigella flexneri serotype 1c derived from serotype 1a by acquisition of gtrIC gene cluster via a bacteriophage
Source: BMC Microbiol. 2016 Jun 27;16:127. doi: 10.1186/s12866-016-0746-z (PMC4924310; doi:10.1186/s12866-016-0746-z)

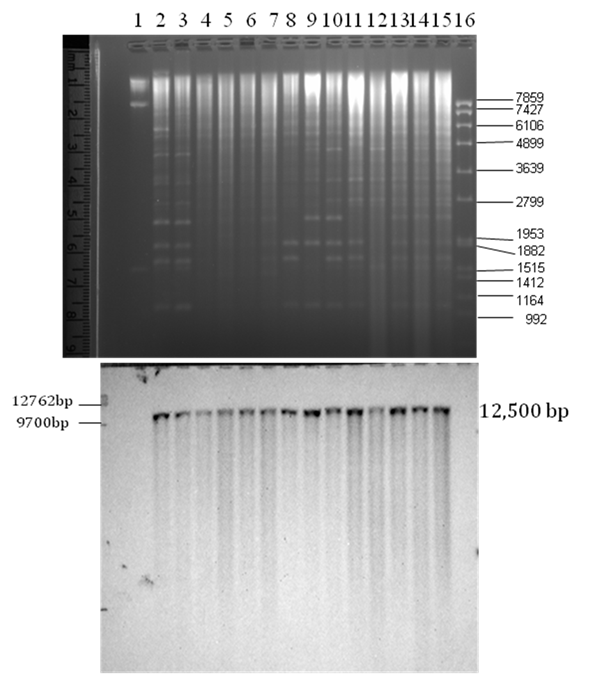


**ii**

**i**

Supplement: Additional file 1: Figure S1. — *Southern blot of BamHI-digested genomic DNA probe with the gtrIC gene. (i) Agarose gel of digested genomic DNA. (ii) Southern blot analysis of digested genomic DNA. Lane 1. SPP-I/XbaI; 2. SFL1502; 3. SFL1684; 4. SFL1685; 5. SFL1686; 6.SFL1687; 7. SFL1575; 8.SFL1576; 9. SFL1578; 10.SFL1579; 11.SFL1556; 12. SFL1557.; 13.SFL1558; 14. SFL1712; 15.SFL1613; 16. Marker SPP-I/EcoRI. (DOCX 240 kb) [file 12866_2016_746_MOESM1_ESM.docx]

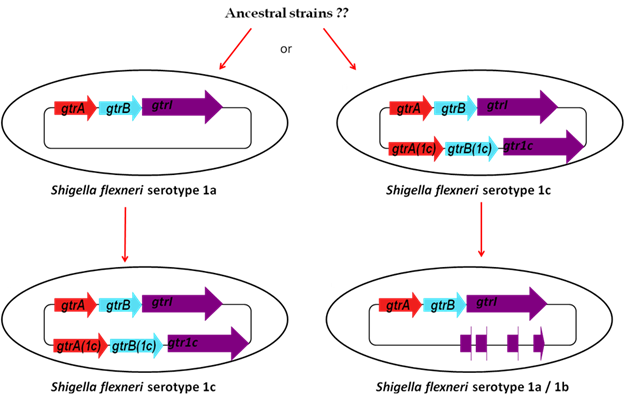


**A**

**B**

Supplement: Additional file 3: Figure S3. — *Schematic diagram illustrating two hypotheses that could potentially explain the evolution of the serotype 1c strain. (A) The gtrIC insertion hypothesis. (B) The deletion of gtrIC hypothesis causing the loss of functional gtrIc modification. (DOCX 115 kb) [file 12866_2016_746_MOESM3_ESM.docx]
