# Supplementary material for: Shigella flexneri serotype 1c derived from serotype 1a by acquisition of gtrIC gene cluster via a bacteriophage
Source: BMC Microbiol. 2016 Jun 27;16:127. doi: 10.1186/s12866-016-0746-z (PMC4924310; doi:10.1186/s12866-016-0746-z)
Supplement: Additional file 2: Figure S2. — *The gtrIC cluster and surrounding 19,147 bp sequence in serotype 1c strain SFL1613 [5]. (DOCX 114 kb) [file 12866_2016_746_MOESM2_ESM.docx]

***gtrIC* cluster**

Insertion relative to pB171

IS629

Isoform

Disrupted IS911

Fragment of ISEhe3

*gtrIC*

isoform

*gtrB_Ic_*

isoform

*gtrA_Ic_*

isoform

Group II intron

IS30

Hypothetical proteins (1-4)

Putative transposase

*narP*

*dsbE*

79 bp of sequence that is 79% identical to Enterobacteria phage phiP27 sequence that includes cos site

*ccmF*

*yejO*

*ccmH*

98% identical to 46625-51313 in *Escherichia coli* plasmid pB171 (AB024946.1)
